# Enhanced physicochemical properties of polydimethylsiloxane based microfluidic devices and thin films by incorporating synthetic micro-diamond

**DOI:** 10.1038/s41598-017-15408-3

**Published:** 2017-11-08

**Authors:** Sidra Waheed, Joan M. Cabot, Niall P. Macdonald, Umme Kalsoom, Syamak Farajikhah, Peter C. Innis, Pavel N. Nesterenko, Trevor W. Lewis, Michael C. Breadmore, Brett Paull

**Affiliations:** 1 ARC Centre of Excellence for Electromaterials Science (ACES), School of Physical Sciences, Faculty of Science, Engineering and Technology, University of Tasmania, Hobart, 7001 Australia; 2 Australian Centre for Research on Separation Science (ACROSS), School of Physical Sciences, Faculty of Science, Engineering and Technology, University of Tasmania, Hobart, 7001 Australia; 3 ARC Centre of Excellence for Electromaterials Science (ACES), AIIM Facility, Innovation Campus, University of Wollongong, Wollongong, NSW 2500 Australia

## Abstract

Synthetic micro-diamond-polydimethylsiloxane (PDMS) composite microfluidic chips and thin films were produced using indirect 3D printing and spin coating fabrication techniques. Microfluidic chips containing up to 60 wt% micro-diamond were successfully cast and bonded. Physicochemical properties, including the dispersion pattern, hydrophobicity, chemical structure, elasticity and thermal characteristics of both chip and films were investigated. Scanning electron microscopy indicated that the micro-diamond particles were embedded and interconnected within the bulk material of the cast microfluidic chip, whereas in the case of thin films their increased presence at the polymer surface resulted in a reduced hydrophobicity of the composite. The elastic modulus increased from 1.28 for a PDMS control, to 4.42 MPa for the 60 wt% composite, along with a three-fold increase in thermal conductivity, from 0.15 to 0.45 W m^−1^ K^−1^. Within the fluidic chips, micro-diamond incorporation enhanced heat dissipation by efficient transfer of heat from within the channels to the surrounding substrate. At a flow rate of 1000 μL/min, the gradient achieved for the 60 wt% composite chip equalled a 9.8 °C drop across a 3 cm long channel, more than twice that observed with the PDMS control chip.

## Introduction

In recent years, the development of polymeric microfluidic devices with integrated functionality has progressed significantly^1,2^. During this period it is also true that polydimethylsiloxane (PDMS) has evolved as the standard material for microfluidic device manufacture, mainly due to its optical transparency, gas permeability, biocompatibility and elasticity^3^. However, one of the deficiencies of PDMS is its low thermal conductivity (0.18 W m^−1^ K^−1^), which equates to poor heat transfer, both from and to fluids held within the micro-channels. As a consequence, for certain temperature sensitive systems, or indeed those involving exo- or endothermic reactions, the internal temperature of the microfluidic platform can vary significantly, which can lead to the deterioration or under-performance of the microfluidic device. Such thermally sensitive systems, wherein thermal management is very important, might, for example, include various lab-on-a-chip (LOC) and micro-electro-mechanical systems (MEMS)^4^, specifically those involving chemical synthesis in microreactors^5^, polymerase chain reactions (PCR)^6^, preconcentration and electromigration based separation techniques^7^ and other highly integrated microsystems^8^.

As early as 1981, Tuckerman *et al*., demonstrated the use of high aspect ratio microchannels for enhanced heat removal^9^. Since this pioneering work, microchannel heat sinks have received considerable attention in many fields. Plastics and composites exhibiting increased thermal conductivity are also in demand to improve heat dissipation and aid stable device performance of high density electronics^10,11^. To-date, PDMS has been modified via the addition of various metallic particles, such as silver (Ag)^12^, gold (Au)^13^, alumina (Al_2_O_3_)^14^, silica^15^, chromium oxide (CrO_2_)^16^ and iron oxide (Fe_2_O_3_)^17^. However, the inclusion of these metallic micro-structures within PDMS is challenging. The low surface energy of PDMS leads to very poor adhesion between the metallic filler and the polymer substrate, which causes structural failures, particularly in the bonding of thin layers^10^. In order to overcome the incompatibility of PDMS and metallic fillers, highly conductive carbonaceous materials are also being investigated^18^. For example, carbon black has been incorporated into PDMS in order to enhance the electrical properties of the composite^19^. Brun *et al*. have also reported the integration of carbon-PDMS nanocomposite electrodes in a PDMS microfluidic chip^20^. This filler preserves PDMS’s processing properties and sustains a high electric field intensities and frequencies without any carbon release. Unger *et al*. have reported doping of carbon black powder above the percolation threshold to create electro-magnetic devices^21^, and carbon nanotubes (CNTs) have also been incorporated in PDMS to improve electrical, rheological and elastic properties in the development of MEMS^22^.

Among these various carbonaceous materials, diamond, specifically detonation nanodiamond and diamond microparticles synthesised at high pressure and high temperature (further called “micro-diamond”), are considered promising candidate fillers due to their hydrophilicity, biocompatibility, non-toxicity, thermal stability, hardness and wear resistance. Several studies have reported the advantages of nano and micro-diamonds as reinforcing enhancers in polymer composites^23,24^. Nakajima *et al*.^25^ developed a thermal interface material (TIM) with high thermal conductivity and electrical insulating property by incorporating diamond and hexagonal boron nitride (h-BN) particles within a silicone matrix. More recently, novel 3D printed composites, containing both nanodiamond^26^ and micro-diamond^27^, have been reported, both displaying enhanced mechanical and thermal properties.

Following these studies, the potential application of micro-diamond-polymer composites in the fabrication of thermally conductive microfluidic devices has emerged. In recent studies, using photo-polymerisation based 3D printing for such applications, the composite material produced were limited to a maximum of 30 wt% of micro-diamonds^27^. This not only limited the obtainable improvement in thermal conductivity, but also limited the composite to print compatible polymers, specifically acrylate based polymers, having limited biocompatibility. Other diamond containing microfluidic devices have been reported, e.g. by Babchenko *et al.*, who fabricated PDMS based microfluidic devices bonded with diamond films^28^, and Karczemska *et al*., who produced diamond based microfluidic devices fabricated through photolithography and etching, with considerable fabrication complexity and cost^29^.

In rapid prototyping of fluidic platforms, the print, cast and peel (PCP) approach, also known as indirect 3D printing, is a promising technique. Herein, we report on such an approach for the rapid fabrication of robust thermally conductive PDMS based microfluidic chips, being doped with micro-diamond at up to 60 wt%. The composite microfluidic chips were fully characterised to reveal their homogeneity, hydrophilicity, flexibility and thermal properties. Enhanced heat dissipation properties were investigated within a microfluidic system at flow rates of up to 1000 μL/ min.

## Results and Discussions

### Homogeneity of PDMS/micro-diamond composites

**Figure 1:**
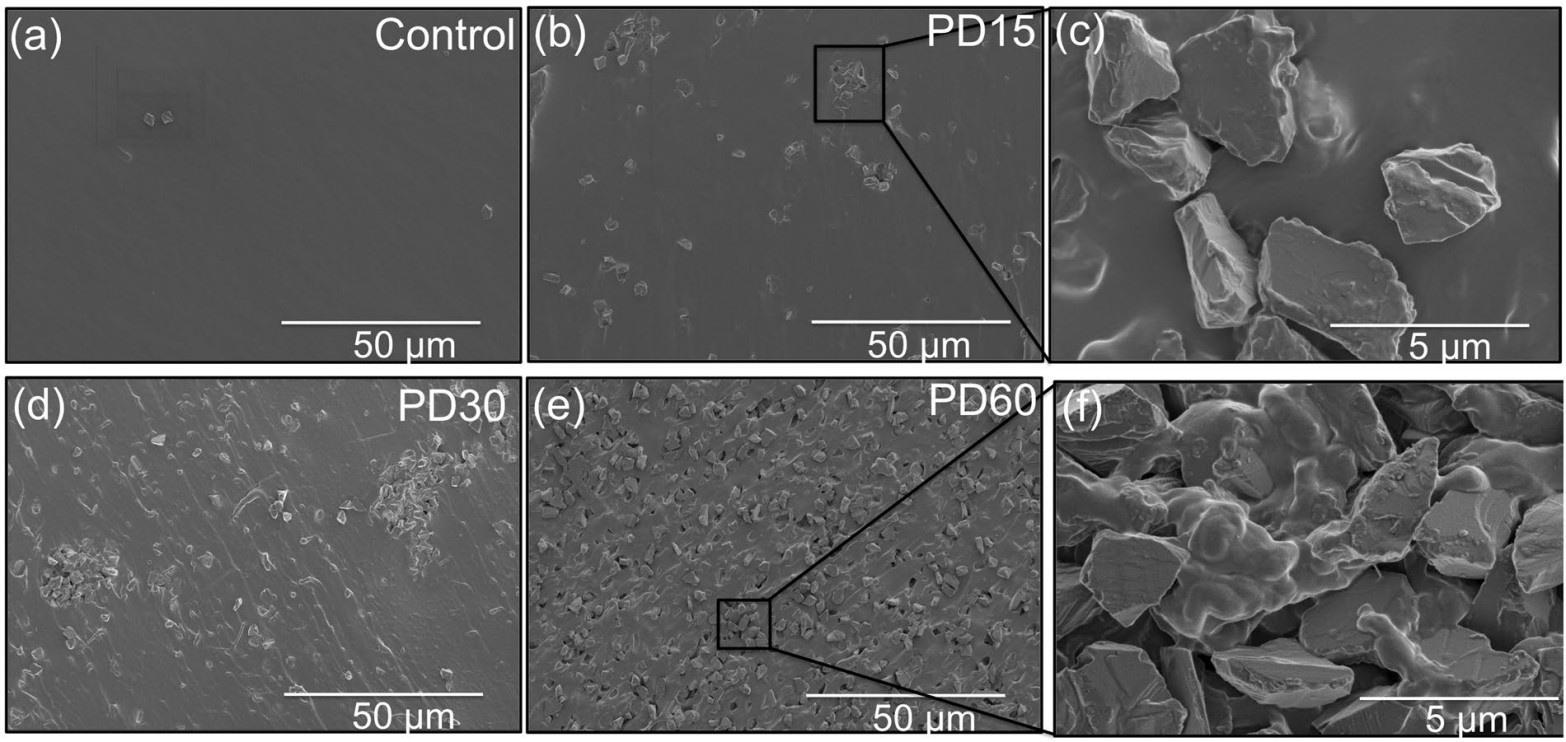
SEM micrographs of a cross-section of microfluidic chip (**a**) PDMS control, (**b**) PD15, (**c**) distribution of micro-diamonds within PD15, (**d**) PD30, (**e**) PD60 and (**f**) dispersion of micro-diamond within polymeric matrix of composite PD60.

The dispersion pattern of micro-diamond on the top surface and cross-sectional surfaces of both control and composite chips containing varying concentrations (15, 30 and 60 wt%) of micro-diamond was observed using scanning electron microscopy (SEM). Images of the top surface of the composite microfluidic chips revealed that the micro-diamond was not present in abundance at the surface, as shown in Supplementary Fig. S1. However, SEM images shown in Fig. 1(a–f) reveal the difference between control and composite material when viewing cross-sections of the various chips. Figure 1(b) and (d) show the more scattered distribution of micro-diamond at lower diamond concentrations, where the micro-diamonds form obvious clusters. At the higher micro-diamond concentrations, as in Fig. 1(e,f), the diamond was homogenously distributed and interconnected.

Contrary to thicker microfluidic chips, SEM micrographs of thin films revealed more evenly scattered distributions of micro-diamond on the top surface of the material. These minor differences were due to different fabrication method used. Applying a spin coating approach to produce the thin film does not provide the depth of PDMS, or time, for the diamond particles to sink below the surface polymer layer, such that their presence throughout the film is maintained. Supplementary Fig. S2 (a–f) reveals the micro-diamond evident at both the surface and evenly distributed throughout the 160 μm deep thin films.

### Hydrophobicity of PDMS/micro-diamond composite

The hydrophobicity of PDMS doped with micro-diamond was investigated by measuring the apparent contact angle (θ°), using the experimental setup shown as Supplementary Fig. S3. The measured contact angle for the PDMS control was 113.7° ± 2.1, which is in close agreement with values reported in the literature^30^. The apparent contact angles for composite microfluidic chips, PD15, PD30 and PD60, measured on the top surface, were 112° ± 2, 116° ± 2 and 113° ± 2, respectively (*n* = 5). These minor changes with respect to the control were not unexpected, as the micro-diamonds were fully encapsulated within the polymeric matrix at the surface, and thus the surface properties of PDMS were not altered. However, when the above experiments were repeated using spin coated films (160 μm thick) for each of the various composites, apparent contact angles of 94° ± 5, 85° ± 4 and 81° ± 5, were obtained, for PD15, PD30 and PD60 films, respectively. The comparison of the contact angle of composite microfluidic chips and thin films is shown in Supplementary Fig. S4. These results suggested the diamond induces a change from an overall hydrophobic material to one which is considerably more hydrophilic in nature.

### Raman spectroscopy of PDMS/micro-diamond composite

**Figure 2:**
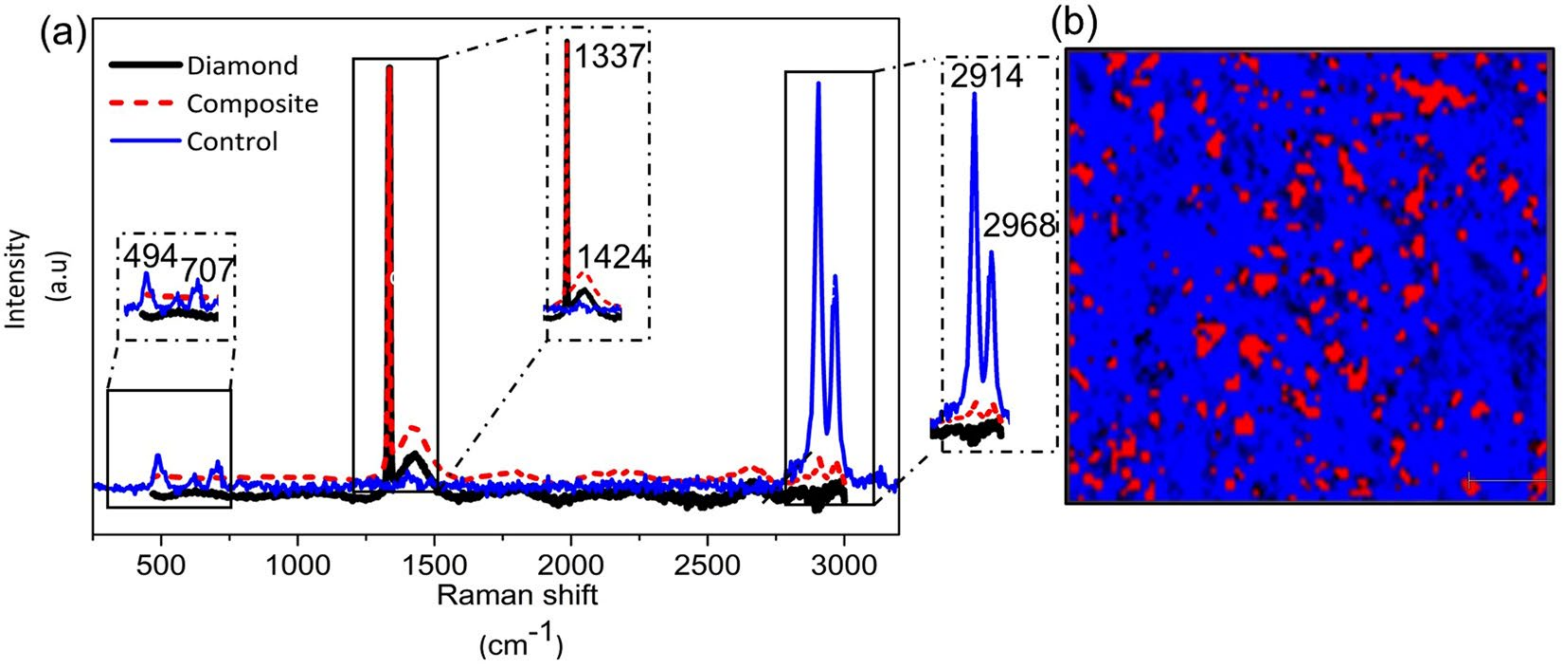
(**a**) Raman spectra of control, pure micro-diamond and composite. (**b**) Raman mapping of PDMS containing micro-diamonds. Red spot indicates micro-diamond distribution based on the peak integration 1319–1348 cm^−1^, while blue indicates polymeric matrix based on the integration 2889–2927 cm^−1^.

Raman spectroscopy was used to characterise the PDMS after the addition of micro-diamond. Figure 2(a) shows the Raman spectra of pure PDMS, micro-diamond itself and the composite material. In the case of pure PDMS, the intense peaks at 2968 cm^−1^ and 2914 cm^−1^ indicate the asymmetric and symmetric stretching of CH groups, respectively. The peak at 707 cm^−1^ represents the symmetric stretching of Si-C, while the peak at 484 cm^−1^ indicates the presence of Si-O-Si^31^. The Raman spectra of pure micro-diamond has a characteristic peak at 1337 cm^−1^ ^32^. The spectra for the composite also exhibits this diamond peak at 1337 cm^−1^. Compared to the control, the stretching of CH (2968 cm^−1^ and 2914 cm^−1^) was significantly decreased with the incorporation of micro-diamond particles. Raman mapping of the composite confirmed the even distribution of the diamond throughout, as shown in Fig. 2(b).

### Elasticity of PDMS/micro-diamond composite

**Figure 3:**
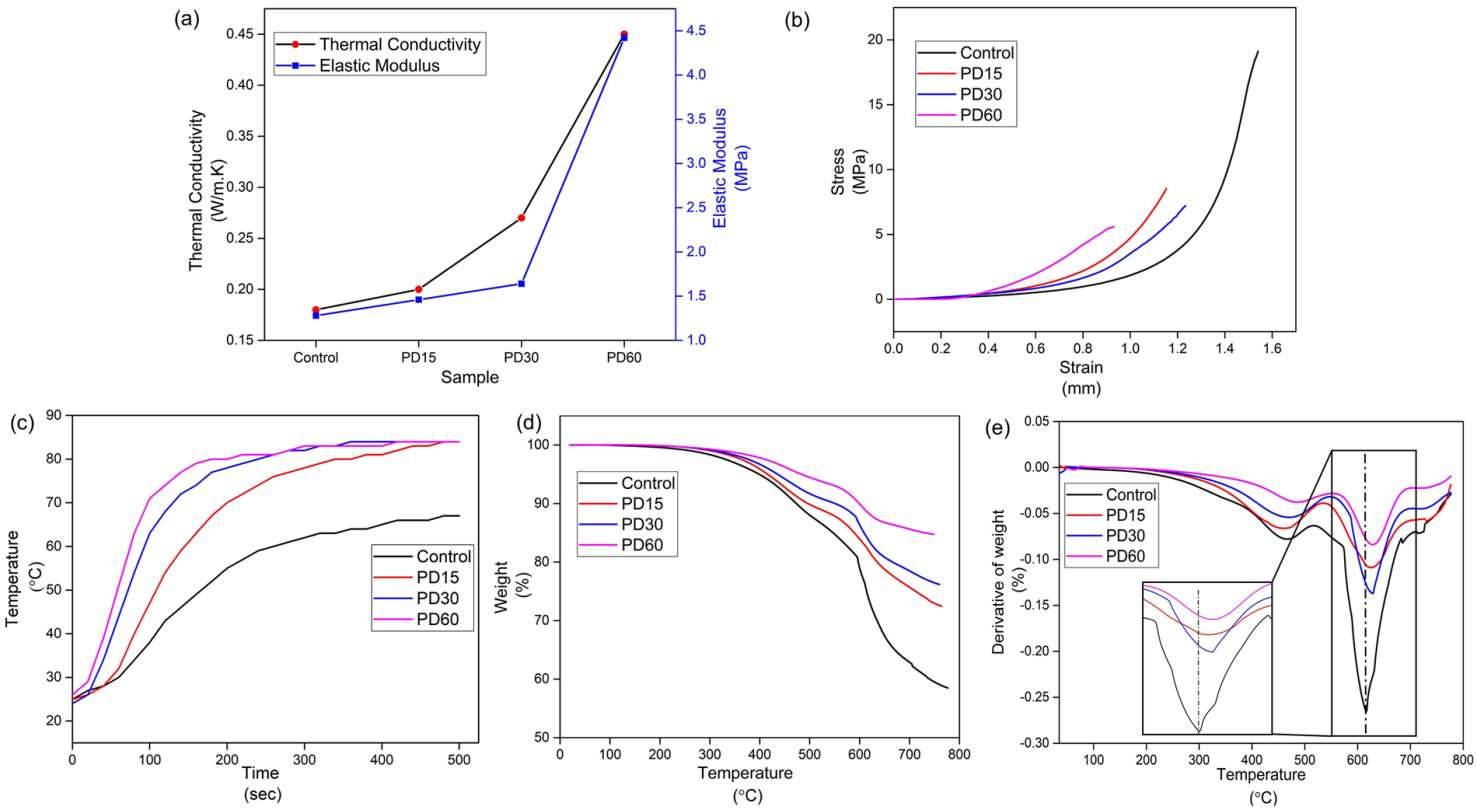
(**a**) Thermal conductivity and elastic modulus for control and composites. (**b**) Stress vs strain curve for control and composite films. (**c**) Temperature variation of top surfaces of the control and composite microfluidic chip, contact heated from below (100 °C) with varying concentration of micro-diamond with respect to time. (**d**) Thermogravimetric curve for control and composite microfluidic chips. (**e**) Derivative of thermogravimetric curve of control and composite microfluidic chips.

The elastic moduli of the control and diamond composites are compared with other composites and polymers in Table 1. The measured elastic modulus for the control is again in agreement with literature values^33^. PD60 displayed a significantly higher (4.42 MPa) elastic modulus as compared to the control, as shown in Fig. 3(a). The low elastic modulus of the control reflects the elastomeric nature of the polymer^34^. PDMS readily losses its elasticity following incorporation of the micro-diamond within the polymer matrix. Figure 3(b) represents stress-strain curve for both control and composite films. It was observed that the composite films (PD15, PD30 and PD60) loss elasticity as doping with micro-diamond increases. However, in all cases the composites demonstrated stress levels higher than 5 MPa.

**Table 1:** Comparison of elastic modulus, thermal conductivity, onset degradation temperature of composite with various loading of fillers and different thermoplastic polymers.

Polymer matrix	Filler loading (wt%)	Elastic Modulus (MPa)	Thermal Conductivity (W/m.K)	Onset degradation temperature (°C)	Reference
PDMS	—	1.28	0.15	284	This study
PDMS	Micro-diamond 15%	1.46	0.20	297	This study
PDMS	Micro-diamond 30%	1.64	0.27	304	This study
PDMS	Micro-diamond 60%	4.42	0.45	310	This study
Polyamide 66	Nano-diamond 3%	—	0.38	—	44
Polyamide 11	Nano-diamond 20%	8000	—		45
PDMS	Al_2_O_3_ 10%	1.94	0.35	—	14
Polyamide 66	—	—	0.24–0.33	—	46
Polyamide 6	—	—	0.28	—	46
Polymethyl acrylate	—	—	0.16–0.25	—	46
Diamond	—	—	900–2320	—	35

### Thermal analysis of PDMS/micro-diamond composite

#### Thermal conductivity

The thermal conductivity of these new polymer-carbon composite materials is a key parameter for many potential applications^35^. From the values shown in Table 1, it is evident that the thermal conductivity of the composite material increases with increasing micro-diamond concentration. With an increase in the concentration of micro-diamond, there is an increase in the number of conductive islands within the matrix and hence an increase in the thermal conductivity of the overall composite. The PD60 composite had a thermal conductivity of 0.45 W m^−1^ K^−1^, which is three times higher than the PDMS control (0.15 W m^−1^ K^−1^), and ~twice higher than other basic polymers (e.g. PMMA, polyamide) listed in Table 1. Recently reported composites of PDMS with both Al_2_O_3_^14^, and nano-diamond^36^ fillers are also listed in Table 1 for comparison, with the PD60 composite described herein providing an ~ 30% and 20% increase in thermal conductivity, respectively.

#### Heat transfer measurements

To measure heat transfer rates, samples of 5 mm thickness were placed upon a heating system set at 100 °C, and the temperature at the top surface was measured every 20 secs. Figure 3(c) shows a plot of the top surface temperature for both control and composite chips as a function of time. The highest temperatures recorded for the control, PD15, PD30 and PD60 were 62, 79.2, 81 and 83 °C, respectively. After reaching these maximum temperatures they all maintained steady state profiles. As is evident from Fig. 3(c), the rise in temperature and time required to reach steady state was considerably improved with an increasing percentage of micro-diamonds, reaching the maximum temperature after only ~3 min for PD60. This confirms the increased thermal conductivity translates into a practical performance parameter, in heat dissipation.

**Figure 4:**
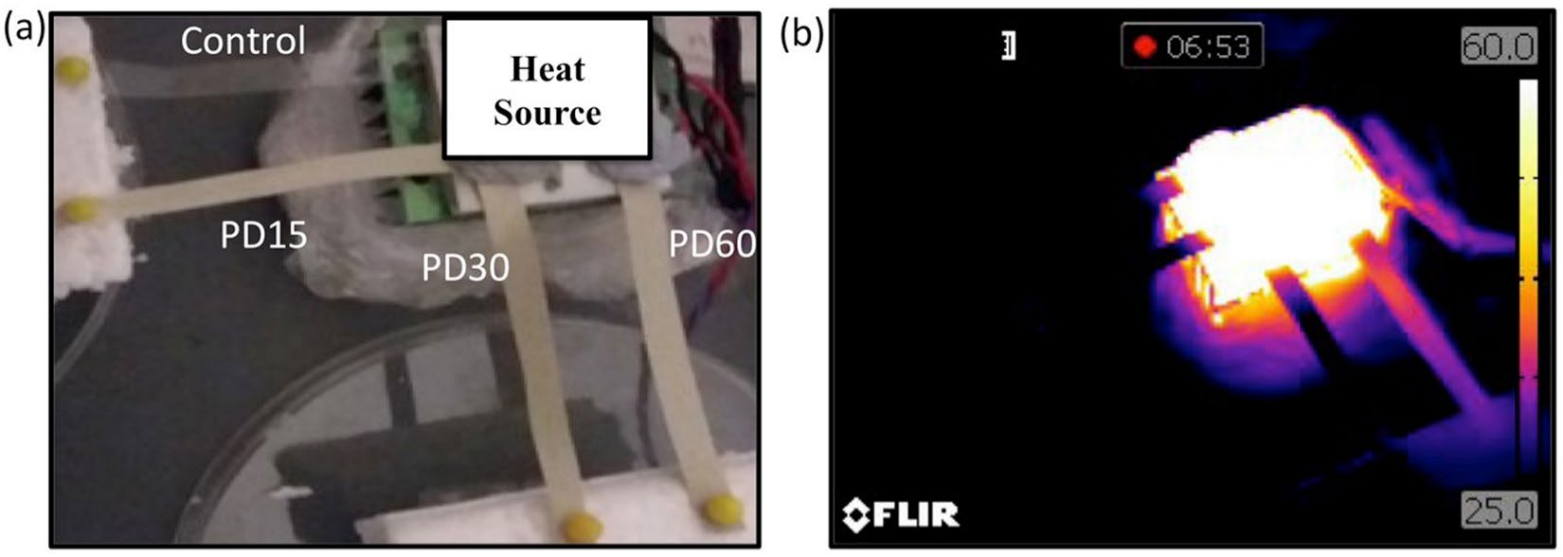
(**a**) Spun coated control, PD15, PD30 and PD60 films attached onto a Peltier module and heated up to 100 °C. (**b**) IR images of control, PD15, PD30 and PD60 films heated at 100 °C from the attached Peltier module.

To demonstrate the improved heat dissipation capability of the composite further, heat transfer across the thin films was measured/observed. Thin films of each composite and the control PDMS, of 75 mm long and ~160 μm thick, were compared by placing one end of film on a Peltier heater module and other end on insulating foam, as shown in Fig. 4(a). The temperature of the Peltier module was raised to 100 °C and the transfer of heat observed using the IR camera. Figure 4(b) shows the dramatic contrast and the success of the PD60 material in dissipating the heat along its length, and transferring heat energy to the insulating foam pad. The real time experiment can be observed as a movie provided within Supplementary Information.

#### Thermogravimetric analysis (TGA)

The impact of micro-diamond on the thermal degradation of PDMS within the various composites was studied using TGA. Figure 3(d) shows the thermal decomposition profiles for the control and composite chips. The degradation onset temperatures measured for the control, PD15, PD30, PD60 were 284, 297, 304, and 310 °C, respectively. Obviously the overall weight loss of each sample decreases with the increasing loading of micro-diamonds, due to its relatively high thermal stability^37^. Figure 3(e) shows the derivative of the thermogravimetric profiles revealing the temperatures where the maximum rate of weight loss occurred. The first peak around 450 °C, was due to the loss of methyl groups in the Si–O backbone. The second peak, at around 620 °C, where maximum weight loss rate is observed, results from the complete degradation of polymeric chain^36^. Clearly the two peak maxima for the three composite materials are shifted towards higher temperatures, compared to that of the PDMS control, confirming their slightly higher thermal stability.

#### Heat dissipation within PDMS/micro-diamond microfluidic chips

**Figure 5:**
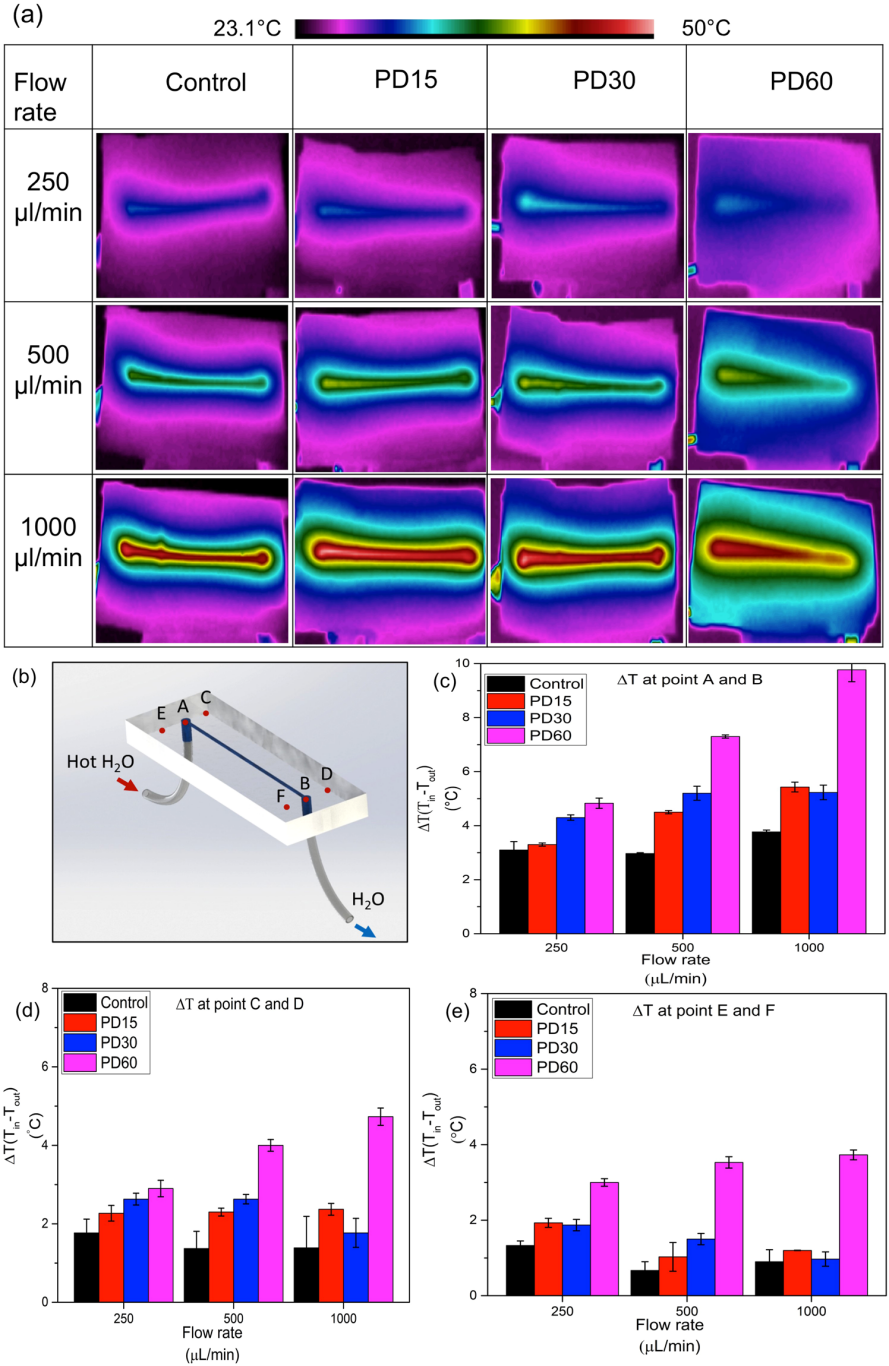
(**a**) IR photographs indicating temperature distribution whilst hot water is passing through the internal channel within the microfluidic chips, prepared using the PDMS control, PD15, PD30 and PD60 materials, at flow rates of 250, 500 and 1000 μL/min. (**b**) Microchannel indicating points where temperature were measured. (**c**) Change in temperature (ΔT) at points A and B, (**d**) points C and D, (**e**) points E and F with respect to flow rate of 250 μL/min, 500 μL/min and 1000 μL/min for control, PD15, PD30 and PD60.

Heated Milli-Q water was pumped through the channels of the control and composite based microfluidic chips at 250, 500 and 1000 μL/min. Representative IR images obtained at each flow rate are shown in Fig. 5(a). The temperature measured at the inlet and outlet of the microfluidic chip can be found in Supplementary Table S1.

As expected the increase in micro-diamond concentration enhances the transfer of heat from the internal channel and across the chip, increasing the temperature of the surrounding area. This results in a greater reduction of the temperature along the length of the channel, achieving greater differences between the inlet and outlet temperatures. A very clear thermal gradient within the channel of the PD60 chip can be seen, highlighting the efficiency of heat transfer out of the fluidic channel. Six points were selected on each microfluidic chip to quantify the variation in temperature. Points A and B on Fig. 5(b) mark the temperature at the inlet and outlet. Points C and D are on the upper side of the inlet and outlet, while points E and F are on the lower side. Plots of temperature change (ΔT) at the inlet and outlet for each flow rate and composite chip are shown as Fig. 5(c–e). The average change in temperature for the control at points A and B, at 250 μL/min, was 3.1 °C ± 0.5, while for PD60 the ΔT was 4.8 °C ± 0.3. The improvement in heat dissipation at 250 μL/min observed with PD60, as compared to the control, was + 56%. At a flow rate of 500 μL/min, ΔT for the control, PD15, PD30 and PD60 were 3.0 ± 0.1, 4.5 ± 0.1, 5.2 ± 0.5 and 7.3 °C ± 0.1, respectively. At 1000 μL/min, the ΔT for PD60 was 9.8 °C within the short 3 cm long channel, more than twice that possible with pure PDMS. A similar trend was observed at points C-D and points E-F. As can be seen in Fig. 5(d) and (e), PD60 achieved approximately 3 times the temperature change compared to the control at flow rates of 500 and 1000 μL/min, while at 250 μL/min ΔT was approximately doubled.

Up to 60% (wt) micro-diamond were successfully dispersed within the polymeric matrix of PDMS. Micro-diamond PDMS composites provided a 200% increase in thermal conductivity, significantly enhancing the dissipation of heat, achieving the optimum dissipation at 60% (wt) micro-diamond. Across a 3 cm long microchannel in a PD60 chip, with a 1000 μL/min flow of water, a ΔT of 9.8 °C was recorded, more than twice that possible with pure PDMS. This proof of concept study offers real potential for a practical solution for the cooling of future electrofluidic, embedded electronics and mechanical-electronic micro systems.

## Materials and Methods

### Materials

A Sylgard® 184 silicone elastomeric kit was purchased from Dow Corning Corporation (Midland, USA). Commercially available Miicraft^+^ resin (BV-003), acrylate based photopolymer, was purchased from Young Optics Inc., Hsinchu, Taiwan. Industrial non-porous high pressure and high temperature micro-diamond powder (2–4 µm) was obtained from Hunan Real Tech Super-abrasive & Tool Co., Ltd. (Changsha, Hunan, China). The micro-diamond powder was purified according to the procedure described elsewhere^38^. Sodium hydroxide, nitric acid and triethoxy (1 H,1 H,2 H,2H-perfluoro-1-octyl) silane were obtained from Sigma Aldrich (Sydney, Australia). Isopropanol was purchased from Ajax Finechem Pty Ltd (Sydney, Australia).

### Template fabrication

**Figure 6:**
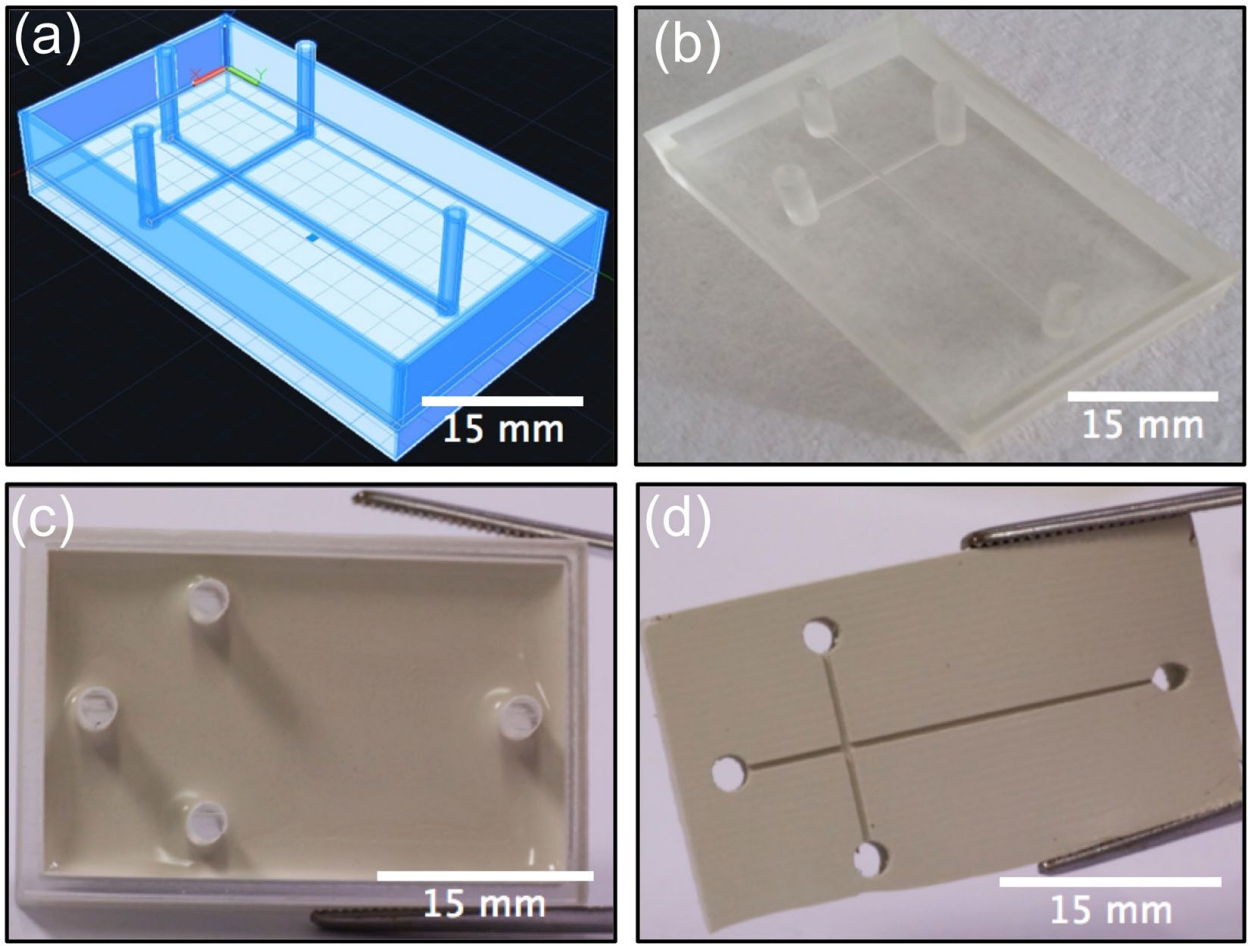
CAD Design and photographs showing the steps involved in the fabrication of diamond PDMS microfluidic chips: (**a**) CAD design of the template with 500 μm channels ending at 8 mm high pillars. (**b**) 3D printed template fabricated using the Miicraft^+^ DLP-SLA 3D printer. (**c**) After silanisation of the template the composite chip is cast. (**d**) Composite chip is peeled from template.

The template was designed with computer aided design (CAD) (Autodesk AutoCAD 2016) software and converted to a STL file with triangle facets using Creation Workshop DataTree3D (Dallas, Texas, USA). The digital 3D model was sliced into 2D cross section layers of 50 μm depth and printed using colourless acrylate based resin BV-003. A Miicraft + (Miicraft, Hsinchu, Taiwan) 3D printer based on Digital Light Processing Stereolithography (DLP-SLA) with resolution of 56 × 56 × 50 μm (XYZ) was used to print the features. The CAD design and printed template are shown in Fig. 6(a) and (b). The dimensions of the microchannel were 500 × 500 μm × 3 cm (width × height × length).

Initial experiments revealed the 3D printed template interfered with the curing of PDMS. This has been postulated to be due to uncured resin left behind on 3D printed template^39^. To overcome this, a post-print surface treatment method was developed to facilitate the curing of PDMS on the template. Comina *et al*., reported a treatment involving coating the template with a protective ink through airbrushing^40^. The group claimed this step required practice to have an optimal finish. Ho *et al*., proposed a more complicated three step method for post-print treatment of templates, involving heating, plasma treatment and surface silanisation^39^. However, it was observed that the heating step involved in the latter method caused cracks in the templates.

A new approach was therefore followed to overcome the disadvantages of the previously reported methods. To this end, a printed template was cured for 5 minutes within the UV chamber of the Miicraft^+^ printer and soaked in isopropanol for 6 hours. The post-print curing and soaking of the template in isopropanol assisted in removing the uncured resin, thought to inhibit the curing of PDMS. Subsequently, the printed template was treated by laboratory corona (air plasma) treatment (BD-20AC, Electro-Technic Products Inc. Chicago, USA) at high power and atmospheric pressure for 1 min and then silanised using triethoxy (1 H,1 H,2 H,2H-perfluoro-1-octyl) silane in a desiccator under vacuum for 3 hours. Silanization was used to provide a hydrophobic fluorinated monolayer on the 3D printed master mould to prevent the PDMS from sticking, allowing the cured PDMS to be peeled off easily (Fig. 6(c) and (d).)

#### Formulation and casting of PDMS/micro-diamond composite device

In order to fabricate any PDMS–diamond composite fluidic chip, the micro-particles must be homogeneously and uniformly distributed within the PDMS matrix. The procedure involved mixing the appropriate amount of micro-diamond powder in the PDMS, followed by sonication for 4 hours. Mixtures of 15, 30 and 60 wt% were prepared and labelled as PD15, PD30, PD60, respectively. The curing agent was then added in a weight ratio of 1:10 to the PDMS-monomer in the mixture. The sample was stirred manually and degassed for 30 min in a vacuum desiccator to remove the trapped air bubbles. The PDMS-diamond composite was then poured onto the 3D printed template as shown in Fig. 6(c). To eliminate any need for hole-punching into the PDMS, pillars of 8 mm height were printed for both inlet and outlet. The composite was cured in an oven at 70 °C for 2 hours, and subsequently peeled from the 3D printed template (Fig. 6(d)). Each composite chip was bonded with a thin film top layer, having dimensions of 30 × 45 mm × 160 μm (width × length × thickness), of identical composition to the underlying material, using laboratory corona treatment^41^.

#### Preparation of composite thin film

**Figure 7:**
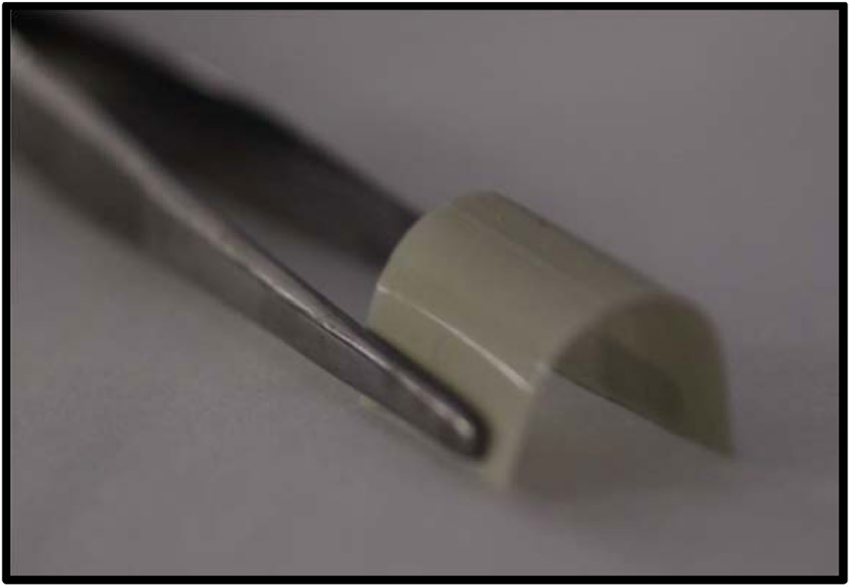
PDMS-micro-diamond composite thin film produced using spin coating approach.

The mixtures of PDMS containing 15, 30 and 60 wt% micro-diamond was sonicated for 4 hours. These mixtures were poured onto a polymethylmethacrylate (PMMA) substrate (50 × 75 × 15mm), placed in a spin coater (WS-650-23NPP, Laurell Technologies, Pennsylvania, USA) and spun under vacuum for 30 sec. The mixture was uniformly coated as a thin film on the substrate. This was then placed in an oven at 70 °C for 2 hours. The film was peeled off from the substrate and used as a top cover for composite chip. Figure 7 shows the resultant composite thin film and microfluidic chip top cover. A pure PDMS cover was used as a control.

Synthetic micro-diamond and PDMS cost $0.275 and $0.119 USD per gram, respectively. In the case of PD60 chips, cost increased approximately by $0.60 USD based on raw material.

### Characterisation of PDMS micro-diamond microfluidic chip and films

Scanning electron microscopy (SEM) imaging was performed with a field emission SEM Hitachi SU70 instrument (Hitachi High Technologies America, USA). Sample cross-sections were cut and placed on carbon tape on Al SEM stubs. Samples were sputtered with a thin (approx. 4 nm) layer of platinum to avoid the accumulation of charge. The dispersion was observed at 1.5 kV.

The hydrophobicity of the PDMS control and each composite were assessed by measuring the contact angles of water droplets placed on their surfaces. The setup used for measuring apparent contact angles is shown in Supplementary Fig. S3. A droplet of 6 μL of Milli-Q water was dispensed from an automated 50 μL eVol XR digital analytical syringe (Trajan Scientific and Medical, Melbourne, Australia) on to the sample surface. The image of the drop on the surface was captured using a Canon DSLR camera (Canon Inc, Tokyo, Japan) with a 70 mm F2.8 EX DG Macro lens (Sigma Corp, Kawasaki, Japan). Apparent contact angles were measured using ImageJ software with an implemented plugin DropSnake^42^. Measurements were carried out at five different points on each chip and average contact angle was measured.

A Renishaw inVia Raman microscope (Wotton-under-Edge, UK) using a monochromatic laser of wavelength 532 nm and Streamline^TM^ imaging technology was used to produce Raman spectra. A cross-section was cut from the sample and placed in a sample holder. The laser was focused onto specific areas with a power of 1.6 mW and an approximate x-y spot size of 1 µm. The scattered light from the surface was collected and directed through the Raman spectrometer using an acquisition time of 10 s and a grating of 1200 lines per mm, resulting in a spectral resolution of about 2.5 cm^−1^. The 2D hyper spectral image was recorded with a step size of 1 µm and an acquisition time of 1 s.

Elasticity was measured on an in-house apparatus, as shown in Supplementary Fig. S5(a). This consisted of a Mitutoyo dial height gauge, which was graduated in 10 µm increments, brass weights and a two decimal place balance. The specimen was cut as shown in Supplementary Fig. S5(b). The upper end was attached to the height gauge arm via a spring clamp. The lower end was attached to brass weights sitting on the balance pan via another spring clamp. The sample was then stretched by raising the height gauge in 1 mm increments and the apparent mass of the brass weights was recorded. The sample was stretched to breaking. Readings of the load applied against extension produced were recorded. The detailed procedure can be found in supplementary information.

Thermal conductivities of the composite chips were measured using a C-therm TCi thermal conductivity analyser (C-Therm Technologies Ltd., Canada), using samples of 2.2 cm length and width and 5 mm thickness. The heat transfer measurements were carried out using a computer controlled Peltier thermoelectric heater/cooler system as detailed elsewhere^43^. The system could operate from room temperature to 100 °C. The composite materials under investigation were placed in the form of blocks having dimension of 22 × 38 × 5 mm (width × length × height) into the system set at room temperature. The loss of heat from the block to the surrounding atmosphere was minimised by enclosing the object within insulation foam during the experiment. A thermocouple was placed on the top surface of the composite to monitor the temperature as the block was heated. Thermal degradation measurements of the composite materials were performed using a Q500 thermogravimetric analyser (TA instruments, USA). 20 mg of each sample was weighed and placed in a platinum crucible. The temperature increased from 35 to 800 °C at a heating rate of 10 °C/min in an atmosphere of nitrogen gas.

The efficiency of heat dissipation within microfluidic chips was studied using the experimental setup shown in Supplementary Fig. S6. A syringe pump Harvard PHD 2000 (USA) was used to pass Milli-Q water through the system. The syringe was connected to an approximately 300 cm long metallic coil with 0.01 mm outer diameter submersed in a hot water bath. Temperature of the bath was maintained at 62 °C using a hot plate fitted with a temperature control sensor. Continuous stirring was provided to ensure uniform heating within the water bath. The heated water was then passed through microchannel at flow rate of 250, 500, 1000 μL/min. Heat dissipation across the external surface was monitored using an infrared camera (FLIR Systems, VIC, Australia). Once a steady state was attained, the temperature at the inlet and outlet was measured by IR camera. Thermal images were taken from underneath the microfluidic chip, which had a 160 μm thick thin film top cover of the same composite formulation. All experiments were carried out in triplicate.

## Electronic supplementary material

Supplementary Information

Video of heat transfer on thin films
