# Supplementary material for: Enhanced physicochemical properties of polydimethylsiloxane based microfluidic devices and thin films by incorporating synthetic micro-diamond
Source: Sci Rep. 2017 Nov 8;7:15109. doi: 10.1038/s41598-017-15408-3 (PMC5678186; doi:10.1038/s41598-017-15408-3)
Supplement: Supplementary file 1 — Supplementary Information [file 41598_2017_15408_MOESM1_ESM.pdf]

Electronic Supplementary Information

Enhanced physicochemical properties of polydimethylsiloxane based microfluidic devices and thin films by incorporating synthetic micro-diamond

Sidra Waheed,[a,b] Joan M. Cabot,[a,b] Niall P. Macdonald,[a,b] Umme Kalsoom[b], Syamak Farajikhah,[c] Peter C. Innis,[C] Pavel N. Nesterenko [a,b], Trevor W. Lewis,[a] Michael C. Breadmore,[a,b] and Brett Paull*[a,b]

[(a)]ARC Centre of Excellence for Electromaterials Science (ACES) School of Chemistry, Faculty of Science, Engineering and Technology, University of Tasmania, Private Bag 75, Hobart, TAS 7001, Australia.

[(b)]Australian Centre for Research on Separation Science (ACROSS), School of Chemistry, Faculty of Science, Engineering and Technology, University of Tasmania, Private Bag 75, Hobart, TAS 7001, Australia.

[(c)]ARC Centre of Excellence for Electromaterials Science (ACES), AIIM Facility, Innovation Campus, University of Wollongong, NSW 2500, Australia.

Corresponding Author:

E.mail: Brett.paull@utas.edu.au; Fax: +61 3 6226 2858; Tel: +61 3 6226 6680

Homogeneity of PDMS/micro-diamond composites

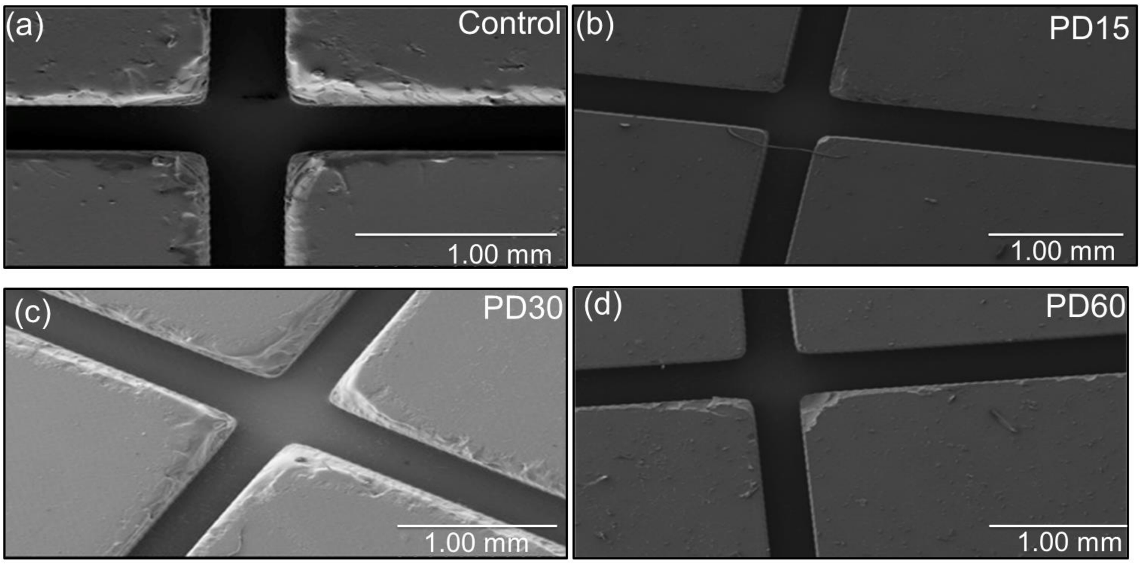

Figure S1 The top surface of control and composite

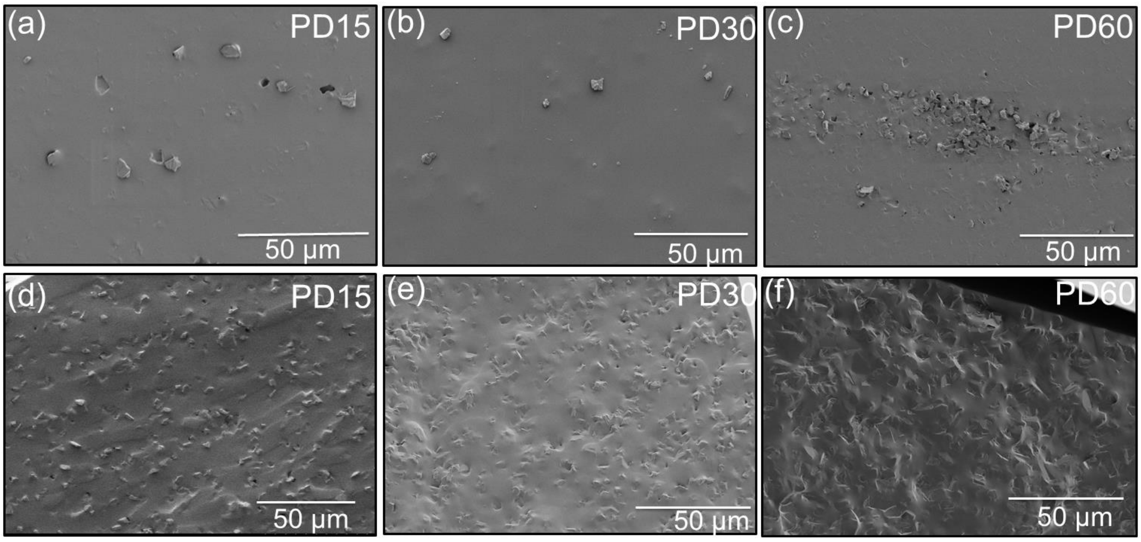

Figure S2 **SEM micrographs of a top surface of films (a) PD15, (b) PD30 and (c) PD60. Cross-section of films (d) PD15, (e) PD30 and (f) PD60.**

Contact angle Measurement

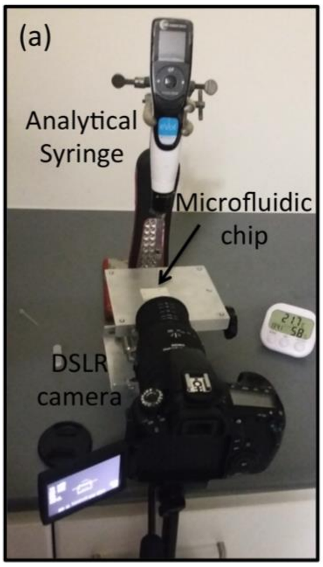

Figure S3: (a) Setup for the measurement of contact angle.

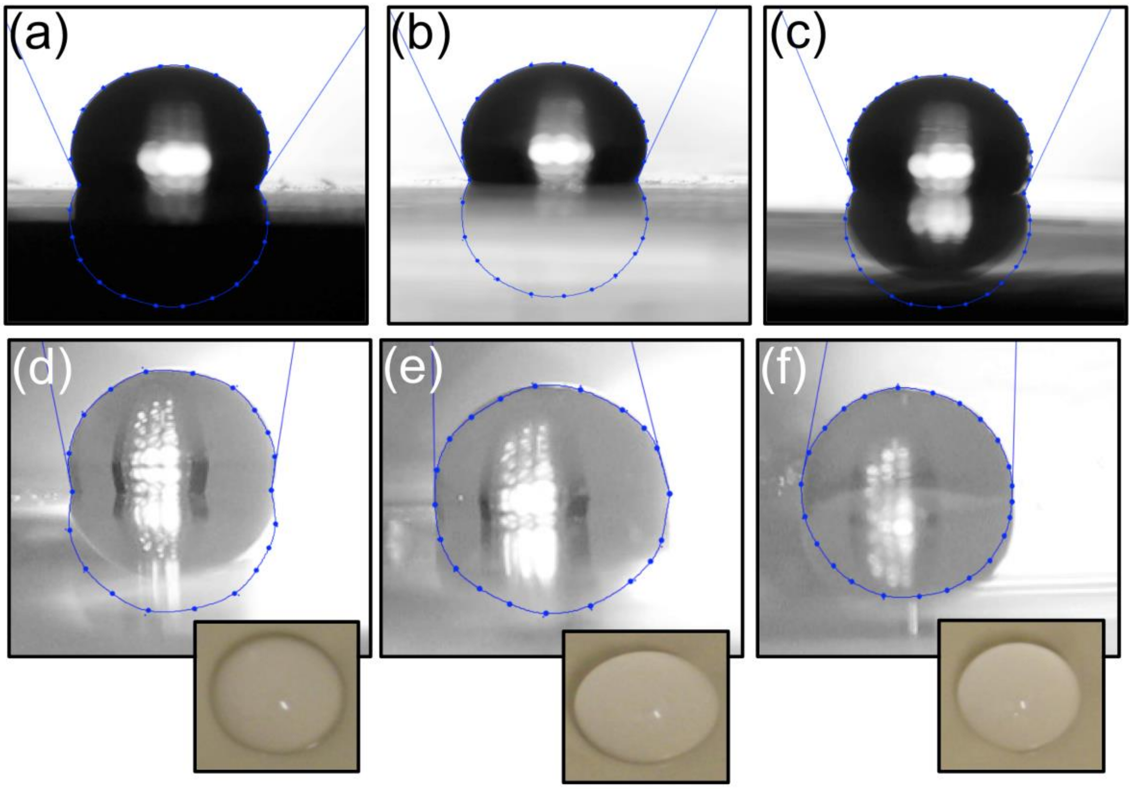

Figure S4. Comparison of contact angle of microfluidic chip (a) PD15, (b) PD30, (c) PD60 and thin films (d) PD15, (e) PD30 and (f) PD60.

Elasticity of PDMS/microdiamond composite

Elasticity was measured on an in-house apparatus, as shown in ESI Fig. S5(a). The specimen was cut as shown in ESI Fig. S5(b). This technique allows load to be resolved to ±0.01 gm force, while extension is measured to ±10 micron resolution. This technique is further automated by adding a Linear Variable Displacement Transducer (LVDT) to the height gauge and a webcam, to read the balance display. The LVDT is connected to an NI6221 Data AcQuisition (DAQ) card and a Microsoft LifeCam to the PC via a USB port. Using LabVIEW (2012), software was designed to monitor the LVDT, and to trigger a photo of the balance display 4 seconds after the LVDT movement had stopped.

Thus the test sequence was:

- Increase the height of the height gauge, stretching the specimen.
- The software waits 4 seconds after the height gauge has stopped moving.
- The webcam takes a picture of the balance reading.
- The software stores the picture of the reading, labeling it with the LVDT reading which is the same value as the height gauge reading.

The software also stores the height reading in a CSV file.

Once the specimen failed, the photos of the balance reading were reviewed and the readings were entered into a CSV file with the corresponding height reading, giving a complete record of extension and load for each specimen.

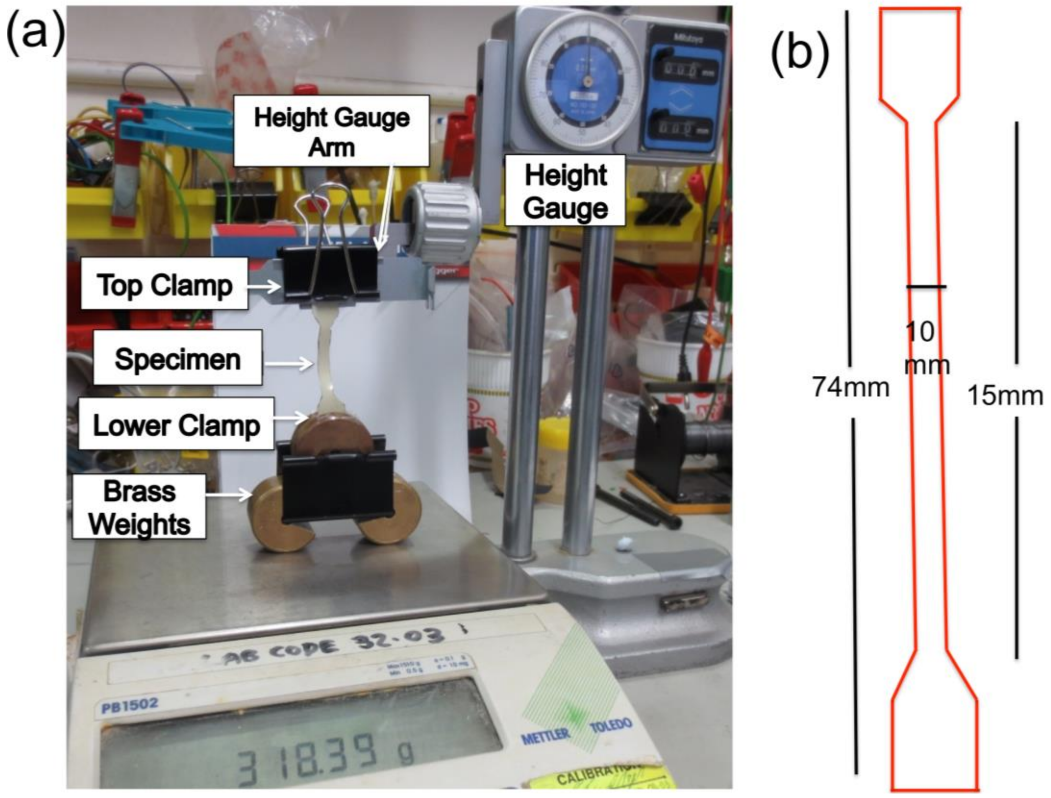

Figure S5: (a) Setup for the measurement of Load and extension **(b)** The geometry of sample

Experimental setup for heat dissipation in a micro-diamond containing microfluidic chip

An experimental setup for heat dissipation measurement in a microfluidic chip is shown in Fig. S6. It consists of syringe pump, hot plate, temperature sensor, stand for holding microfluidic chip, microfluidic chip and IR camera.

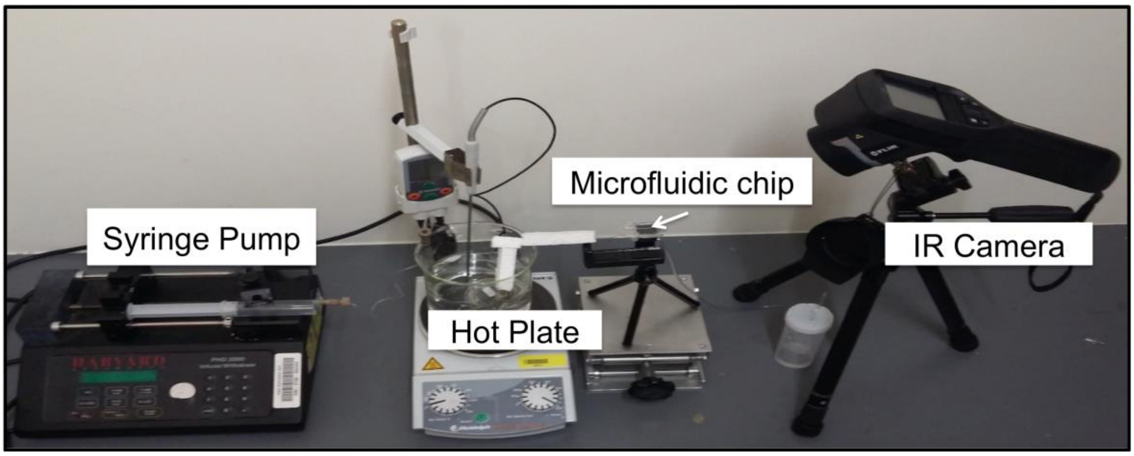

Figure S6: Photo of an experimental setup for thermal measurements of microfluidic platform with flow of Milli-Q water using IR camera.

Sample	Inlet	Outlet	Inlet	Outlet	Inlet	Outlet

	Temperature (°C)	Temperature (°C)	Temperature (°C)	Temperature (°C)	Temperature (°C)	Temperature (°C)
**Flow rate**	250 µl/min		500 µl/min		1000 µl/min	
**Control**	32.3	29.2	38.5	35.5	46.13	42.3
**PD15**	32.0	29.83	40.6	35.9	47.3	41.9
**PD30**	32.7	28.4	39.6	34.4	45.8	40.6
**PD60**	32.1	27.3	39.3	32	46.7	36.9

Thermal performance of PDMS/micro-diamond chip under various flow rates

Table S1: The temperature at inlet and outlet of the microfluidic chip at different flow rate
